# Exploring Machine Learning Approaches for Decision Support in Neoadjuvant Therapy of Locally Advanced Rectal Cancer

**DOI:** 10.32604/or.2026.074385

**Published:** 2026-03-23

**Authors:** Eshita Dhar, Muhammad Ashad Kabir, Divyabharathy Ramesh Nadar, Li-Jen Kuo, Jitendra Jonnagaddala, Yaoru Huang, Mohy Uddin, Shabbir Syed-Abdul

**Affiliations:** 1 Graduate Institute of Biomedical Informatics, College of Medical Science and Technology, Taipei Medical University, Taipei, Taiwan; 2 International Center for Health Information Technology, College of Medical Science and Technology, Taipei Medical University, Taipei, Taiwan; 3 School of Computing, Mathematics and Engineering, Charles Sturt University, Bathurst, Australia; 4 CGD Health Pvt Ltd., Mumbai, India; 5 Division of Colorectal Surgery, Department of Surgery, Taipei Medical University Hospital, Taipei, Taiwan; 6 Discipline of General Practice, School of Clinical Medicine, Faculty of Medicine, UNSW Sydney, Kensington, Australia; 7 NMC Royal Hospital, Khalifa City, Abu Dhabi, United Arab Emirates; 8 Research Quality Management Department, King Abdullah International Medical Research Center, King Saud bin Abdulaziz University for Health Sciences, Ministry of National Guard Health Affairs, Riyadh, Saudi Arabia; 9 School of Gerontology and Long-Term Care, College of Nursing, Taipei Medical University, Taipei, Taiwan

**Keywords:** Machine learning, chemoradiotherapy, rectal cancer, treatment response, predictive modelling

## Abstract

**Objectives:**

Decisions regarding CT after nCCRT for locally advanced rectal cancer (LARC) are challenging due to limited evidence guiding treatment. This study aimed to (i) evaluate the predictive performance of machine learning (ML) models in patients treated with neoadjuvant concurrent chemoradiotherapy (nCCRT) alone vs. those receiving nCCRT plus chemotherapy (CT), (ii) identify features associated with treatment improvement, and (iii) derive ML-based thresholds for treatment response.

**Methods:**

This retrospective study included 409 patients with LARC treated at three affiliated hospitals of Taipei Medical University. Patients were categorised into two groups: nCCRT alone followed by surgery (*n* = 182) and nCCRT plus additional CT (*n* = 227). Thirty-four baseline demographic, tumor, and laboratory variables were analysed. Four ML algorithms (K-Star, Random Forest, Multilayer Perceptron, and Random Committee) were evaluated, while five feature-ranking algorithms identified influential attributes among improved patients across both treatments. Decision Stump and AdaBoostM1 were applied to derive threshold-based patterns.

**Results:**

K-Star achieved the highest accuracy for nCCRT alone (80.8%; AUC = 0.89), while Random Committee performed best for nCCRT plus CT (77.3%; AUC = 0.84). Clinical N stage (cN) ranked highest, followed by Sodium (Na), Glutamic pyruvic transaminase, estimated glomerular filtration rate, body weight, red blood cell count, mean corpuscular hemoglobin concentration, and blood urea nitrogen. Threshold patterns suggested that CT-related improvement aligned with higher lymphocyte percentage and lower platelet distribution width, whereas nCCRT-only improvement aligned with elevated eGFR, GPT, and cN = 2. Conclusions: ML-based analysis identified key predictors and demonstrated good model performance, supporting individualised post-nCCRT chemotherapy decisions.

## 1 Introduction

Colorectal cancer (CRC) is one of the most prevalent cancers in both men and women and is a leading cause of mortality worldwide. The incidence of CRC has been increasing, especially in industrialised and emerging economies. This is due to the adoption of and shift into lifestyle patterns characterised by reduced physical activity, greater sedentary time, and increased consumption of processed or calorie-dense foods, red meat, alcohol, and tobacco [1,2]. There were an estimated 1.93 million newly diagnosed cases of CRC and 0.94 million mortalities due to CRC in 2020 worldwide. Based on projections of ageing, population growth, and human development, new cases of CRC are predicted to reach 3.2 million globally by 2040, with China and the US projected to be the frontliners for the highest number of cases over the next two decades [2]. In addition, the incidence of early onset CRC has increased in high-income countries over the past few decades and is projected to steadily increase by approximately twofold by 2030 [3]. These expanding trends are a global health concern and have raised an alarm for the prevention and control of CRC. Modifiable risk factors such as obesity, unhealthy diet, sedentary lifestyle, smoking, and high alcohol consumption are driving forces for the incidence of and deaths due to CRC [4,5]. Advancements in early detection, screening, and treatment options have played a prominent role in reducing the mortality caused by CRC. The treatment modalities for rectal cancer differ slightly because of the anatomical structure of the rectum, especially for locally advanced rectal cancer (LARC), which is characterised by deep tumor invasion or regional lymphadenopathy. The conventional surgical approach may result in permanent colostomy of the stomach, which results in a dismal and inconvenient daily life. The current recommendation for neoadjuvant concurrent chemoradiotherapy (nCCRT) before surgery for LARC is to preserve the anal sphincters and enhance patients’ quality of life [6].

Since their inception, technological advancements in artificial intelligence (AI) and machine learning (ML) have significantly influenced the healthcare sector by providing effective and high-quality systems and services. With the development and potential of AI in healthcare, ML has been widely used and has shown promising prospects for disease prediction, treatment, and prevention [5]. ML has transcended traditional statistical and research limitations, offering unique opportunities and analytic capabilities to solve healthcare challenges and improve delivery. Currently, the voluminous amount of high-quality CRC data collected from health information systems can be leveraged using evidence-based ML models. These models can enhance the knowledge of CRC prediction at an early stage [7]. ML algorithms have been extensively utilised for learning trends, producing insights into patterns and behaviors, and making predictions based on previously unseen cases using patient data [8]. ML models can help obtain a clear and faster diagnosis of colorectal cancer in the initial stage, increase the success of treatment, and reduce mortality rates due to this cancer. This prediction of the presence of colorectal cancer in the early stages and identification of the cancer stage are crucial for determining treatment plans and improving the lifestyle of cancer patients [9].

Numerous studies have demonstrated the importance of ML-based diagnostic systems in supporting clinicians in making accurate diagnostic and therapeutic decisions for various types of cancers. A recent study showed multimodal approaches combining whole slide images with clinical and molecular data improves accuracy in CRC prediction [10]. Several systematic and narrative review articles [8,9,11,12] have summarised studies utilising ML models, specifically in the field of CRC. Previous research has explored the use of diverse data sources, including laboratory test results, histopathological data, and clinical text, for the identification and prognosis of colorectal cancer (CRC). For instance, a Taiwanese study derived a tumor aggression score as a prognostic factor to predict the stage of tumors and survival via histopathological data among colon cancer patients [13]. Based on patient demographics and laboratory test results, a U.S. community-based study presented a CRC detection model for the reliable identification of patients in curable stages of CRC, revealing that it performed better than a single Hb threshold screening [14]. A Chinese retrospective study [15] extracted the laboratory data of patients with CRC and healthy individuals from electronic medical records and utilised various ML models to identify late-stage CRC. Another study from China applied Swin Transformer models to predict microsatellite instability and key biomarkers in CRC from H&E-stained images, achieving state-of-the-art performance even with limited data [16]. Another Italian study utilised colorectal cancer case data to demonstrate the feasibility and potential prognostic value of histopathological features derived from ML models for survival of patients with colon cancer [17]. Another study leveraged large language models to deidentify and normalize sensitive electronic health record data, ensuring privacy and consistency for downstream AI applications [18]. Previous research has shown the potential of ML in predicting the use of chemotherapy (CT) and evaluating the efficacy of CRC treatment [5]; however, to the best of our knowledge, no further investigations focusing on preoperative scenarios have been conducted. Currently, neoadjuvant concurrent chemoradiotherapy (nCCRT) is the established standard of care for LARC patients. nCCRT may increase the success of the operation, reduce its side effects, and potentially alter postoperative treatment if the tumor reaches pathological complete remission (pCR).

However, the current clinical dilemma for LARC is whether patients should undergo further CT after nCCRT or before surgery. A 2015 meta-analysis suggested that there is no benefit for additional CT [19], whereas a study in 2018 using total neoadjuvant therapy (TNT) [20] and the PRODIGE 23 trial using FOLFIRINOX after nCCRT [21] suggested better progression-free survival than nCCRT alone. However, no improvement in surgical outcomes or local control was noted, and the lower compliance rate of the additional stronger CT increased concerns about more severe side effects, and critically, these trials often lacked demonstrated improvement in pathological complete response rates or local control, reinforcing the clinical uncertainty. In clinical decision-making, some surgeons may opt for a compromised CT regimen with a shorter duration and less toxic chemotherapy, whereas others may prefer to administer nCCRT alone initially and provide adjuvant CT only after surgery, as it has the least risk and side effects. Given the promising potential of ML approaches for clinical decision-making support, we aimed to investigate predictive models to assist in determining which patients may benefit from additional CT.

The objectives of the study are to (i) develop ML models predicting outcomes in patients treated with nCCRT alone and in those receiving nCCRT plus additional CT; (ii) identify baseline attributes associated with favorable outcomes across the two treatment regimens; iii) explore threshold-based clinical and biochemical features that differentiated patients who improved after nCCRT alone from those who improved after nCCRT with additional CT.

## 2 Material and Methods

### 2.1 Study Design

This study was approved by the Taipei Medical University Joint Institutional Review Board (TMU-JIRB no. N202006049), which also granted a waiver of informed consent because the analysis used de-identified retrospective clinical data and posed no risk to patient privacy or safety. The study was conducted in accordance with the Declaration of Helsinki

All centers used shared TMU institutional standards for diagnosis, treatment, data documentation, and pathology reporting, which helped reduce variation in recording practices. Data consistency was further supported through harmonised electronic medical records and routine institutional quality-control procedures. All analyses were conducted using de-identified data. The dataset covers the period from January 2016 to December 2022. Our study population included patients with newly diagnosed locally advanced rectal cancer (LARC). These patients were categorised into two groups: (1) those who received neoadjuvant concurrent chemoradiotherapy (nCCRT) followed by surgery, and (2) those who received nCCRT with additional chemotherapy prior to surgery.

**Figure 1:**
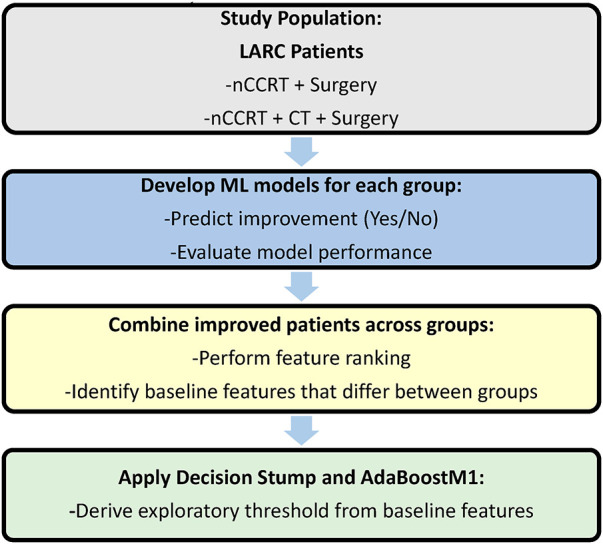
Study design and analytical workflow

The Joint Tumor Board of Colorectal Cancer from three hospitals provided treatment guidelines for rectal cancer. The guidelines recommend nCCRT before curative surgery for patients who are newly diagnosed with rectal adenocarcinoma, with either T3–4 or nodal positive status. The nCCRT regimen consisted of standard fractionation radiotherapy (RT) (50 Gy/25 fractions or 50.4 Gy/28 fractions), whereas concurrent chemotherapy included oral capecitabine (1250 mg/m^2^ twice daily) or intravenous 5-fluorouracil-based regimens (2400–3000 mg/m^2^ continuous IV infusion over 46–48 h). After completing nCCRT, a watch-and-wait observation was scheduled, although oral or intravenous CT was not mandatory during this period and was based fully on clinical judgment and individual preferences. Curative surgery for rectal cancer, either robotic or laparoscopic low anterior resection (LAR) or abdominal-perineal resection (APR), was arranged 8–12 weeks after the completion of nCCRT. A full pathological review was conducted after surgery, and cancer staging was based on the TNM system of the American Joint Committee on Cancer Staging, 8th edition. Pathological evaluation followed the standard institutional protocol, in which the reporting pathologist drafted the report and the head of pathology verified and signed it. Fig. 1 illustrates the study design and analytical workflow of the research.

### 2.2 Study Criteria

The inclusion and exclusion criteria are summarised in Table 1.

**Table 1:** Eligibility criteria for patient selection in the study

Inclusion criteria	Exclusion criteria
Age >18 years at the time of cancer diagnosis.	Deviation from the recommended nCCRT protocol before surgery (incomplete nCCRT, with other C/T regimens, different fractions of R/T, or early or delayed surgery for any cause.
Diagnosis of rectal adenocarcinoma with proof of biopsy.	Surgery was performed outside the three participating hospitals.
Full evaluation of clinical staging, including CT and/or MRI resonance imaging.	No curative surgery or incomplete pathological review after surgery.
Recommended for nCCRT before surgery by the colorectal tumor board.	Diagnosis of another cancer during the treatment period.
No previous history of colorectal cancer.	
No synchronous treatment for other cancers.	

Note: CT: Chemotherapy, nCCRT: Neoadjuvant concurrent chemoradiotherapy, MRI: Magnetic resonance imaging, C/T: Chemotherapy, R/T: Radiotherapy.

Patients receiving treatment for other cancers were excluded because additional malignancies and their therapies could influence tumor response and interfere with outcome assessment. Patients who underwent surgery outside the three participating centers were also excluded to ensure consistency in surgical procedures, pathology evaluation, and staging across the cohort.

### 2.3 Data Analysis and AI Model Development

Additional chemotherapy (CT) before surgery may cause toxicity, poor compliance, and greater surgical risk; deciding whether to add CT after nCCRT is a critical challenge. The decision to receive additional CT is mostly based on clinical judgment, and the specific CT regimen, duration, and toxicity varied based on physician discretion, contributing to the clinical heterogeneity addressed by this study. In this study, we developed separate ML models to predict the likelihood of improvement for patients treated with nCCRT alone and those treated with nCCRT plus CT. The current modelling framework directly addresses our first objective by estimating the likelihood of improvement under each treatment pathway. These models can flag patients who are likely to improve sufficiently with nCCRT alone, thereby sparing them from toxic additional chemotherapy, and identify patients who are less likely to improve with nCCRT alone, for whom chemotherapy may be warranted. While exploratory, this dual-model approach demonstrates the potential of ML to support individualised decision-making in LARC. Deidentified data from each hospital were used for centralised analysis. Pre-processing involved excluding three variables with high and inconsistent missingness: mean platelet volume, RDW standard deviation, and plateletcrit, each with approximately 47% missing data. Removing these variables did not affect the model’s clinical interpretability because they are not routinely used in treatment decision-making for rectal cancer, and the remaining predictors covered all major haematological, biochemical, and tumour-related domains relevant to this study.

After this step, the final dataset contained complete data for all predictors used in model development. Continuous variables were kept in their original clinical units because the algorithms used do not require normalization. We deployed four ML models using K-Star (K*), random forest (RF), random committee (RC), and multilayer perceptron (MLP).

RF and RRC are ensemble-based models. RF combines several decision trees to increase prediction stability, while RC reduces variance by averaging the results of multiple randomly selected base learners. K-Star is an instance-based learning technique that classifies a new case by finding the most similar cases in the data and using their outcomes to guide the prediction. Lastly, MLP is a neural network that uses interconnected layers to model nonlinear relationships. Overall, these models offer a fair assessment of ensemble, instance-based, and neural methods.

To prevent potential bias in classifier learning and to ensure equal representation of the improved and non-improved outcome categories, we applied the Synthetic Minority Oversampling Technique (SMOTE) separately within each treatment group before model training. All models were evaluated using 10-fold cross-validation (CV) to minimise overfitting. In each round, nine folds were used for training and one fold for testing. Oversampling was applied only to the training folds, while the test folds retained their original class distributions.

Model performance was evaluated using accuracy, precision, recall, F1-score, and the area under the ROC curve, based on true positives, false negatives, false positives, and true negatives. Performance metrics are reported with 95% confidence intervals (CI), with accuracy CIs calculated using Wilson’s method and AUROC CIs estimated using the Hanley-McNeil approach.

### 2.4 Feature Selection

Five attribute ranking algorithms (Information Gain, ReliefF, OneR, CfsSubset, and Correlation) were applied to the combined cohort of improved patients (*n* = 217), along with baseline demographic, clinical, and laboratory variables, to identify the most relevant variables for model development. Only patients who demonstrated improvement were included, enabling the ranking methods to focus on variables specifically distinguishing those who benefited from treatment in either group. This step directly addressed the second objective of identifying baseline variables associated with treatment improvement. The outputs of the five ranking methods were reviewed together to compare the relative ordering of variables, and the final feature set was determined on this comparative assessment. Thereafter, Decision Stump and AdaBoostM1 were applied to the combined cohort of patients who had improved. Within the improved cohort (*n* = 217), we employed these two simple supervised models to suggest transparent cut-offs in baseline variables that distinguish patients who improved after nCCRT alone from those who improved after nCCRT plus chemotherapy. A decision stump (one-split tree) selects a single variable and threshold to form an if-then rule. AdaBoostM1 builds many such stumps and combines their weighted votes, yielding additional model-derived cut-offs. These rule-based models support our third objective of exploring simple baseline thresholds associated with treatment response. The thresholds were derived to provide interpretable summaries of baseline characteristics and were not intended as predictive decision rules.

## 3 Results

### 3.1 Cohort Characteristics

**Figure 2:**
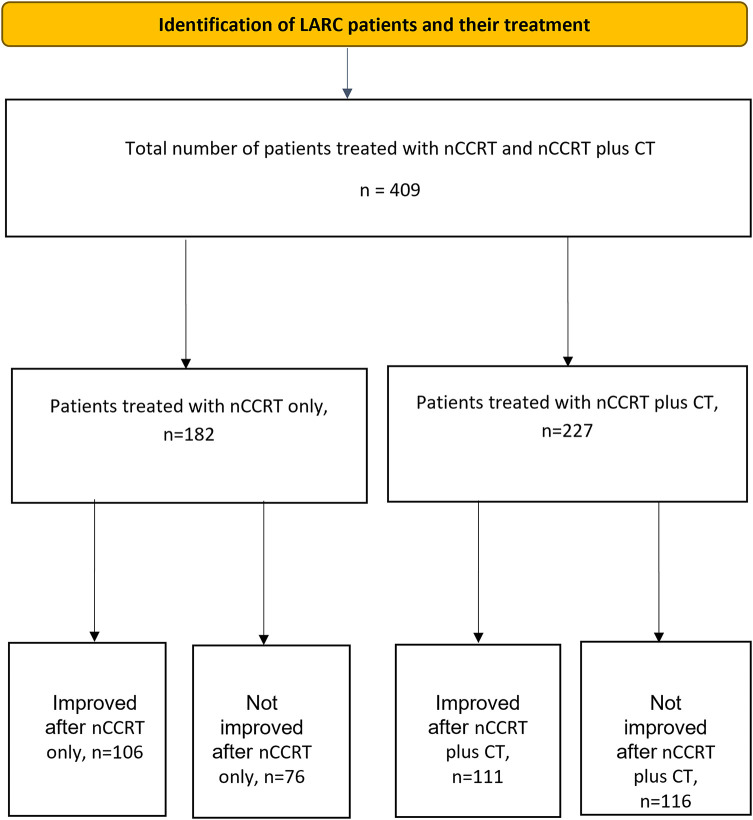
Flow diagram showing the number of patients, treatment allocation, and outcomes. Note: LARC, locally advanced rectal cancer; nCCRT, neoadjuvant concurrent chemoradiotherapy; CT, chemotherapy

The distribution of patients across the two treatment groups is shown in Fig. 2. A total of 409 patients, ranging in age from 23 to 85 years, were included in the de-identified analysis, with 267 being male. Among these patients, 372 had T3–4 tumors, whereas 317 had regional lymphadenopathies classified as N1–2. None of the patients had any metastatic lesions.

All patients completed nCCRT and/or additional CT and underwent curative surgery (LAR or APR), as scheduled. None of the patients underwent delayed surgery owing to the severe side effects of neoadjuvant treatment. Of the 409 patients with LARC (mean age, 60.1 years; range, 23–85), 182 patients received nCCRT alone and 227 received additional CT after nCCRT. Overall, 217 patients (53.1%) demonstrated tumor downstaging, while 192 (46.9%) did not.

Baseline features are summarised in Table 2 and included demographics, haematological indices, platelet indices, renal function markers, liver function markers, differential leukocyte counts, electrolytes, and the tumor marker CEA. Attributes with high missing values (numbers 17, 18, and 20; corresponding to mean platelet volume, RDW standard deviation, and plateletcrit) were dropped from further analysis due to high missing data values (approx. 47%).

**Table 2:** Basic characteristics and features of the patients included in the study

No.	Attributes	Min values/Frequencies	Max numeric	Mean (Std. Dev)	Missing N (%)
1	Age, years	23	85.4	60.1 (11.9)	0 (0%)
2	Sex				0 (0%)
		Male, *n*	267		
		Female, *n*	142		
3	Weight (kg)	37	185	142.3 (41.6)	1 (0%)
4	Clinical T stage (cT)				0 (0%)
		0		1	
		1		2	
		1a		1	
		2		33	
		3		311	
		4		15	
		4a		16	
		4b		30	
5	Clinical N stage (cN)				0 (0%)
		0		92	
		1		57	
		1a		31	
		1b		71	
		1c		1	
		2		38	
		2a		67	
		2b		52	
6	Clinical M stage (cM)				0 (0%)
		0		365	
		1		11	
		1a		20	
		1b		9	
		1c		1	
		x		3	
7	WBC (10^3^/µL)	2.52	21.2	7.6 (2.4)	8 (2%)
8	RBC (10^6^/µL)	2.08	6.7	4.4 (0.6)	9 (2%)
9	Hemoglobin (HGB, g/dL)	4.4	17.3	12.8 (2.1)	8 (2%)
10	Hematocrit (HCT, %)	15.7	49.8	37.9 (5.6)	9 (2%)
11	Mean corpuscular volume (MCV, fL)	54.7	110.9	85.9 (9.1)	9 (2%)
12	Mean corpuscular hemoglobin (MCH, pg)	15.2	38.7	28.9 (3.8)	9 (2%)
13	MCHC (g/dL)	27.8	36.4	33.5 (1.3)	9 (2%)
14	RDW (%)	12	294	15.7 (16.6)	40 (10%)
15	Platelet count (PLT, /µL)	83	678	270.7 (91.2)	8 (2%)
16	Mean platelet volume (MPV, fL)	6.31	12.4	8.45 (1.2)	192 (47%)
17	RDW-SD (fL)	33.3	69.1	42.6 (4.8)	191 (47%)
18	Platelet distribution width (PDW, fL)	8.2	19.4	15.7 (2.3)	105 (26%)
19	Plateletcrit (PCT, %)	0.09	0.45	0.22 (0.07)	193 (47%)
20	Neutrophil (%)	28.4	93.9	65.2 (9.14)	28 (7%)
21	Lymphocyte (%)	4	67.1	24.1 (8.2)	28 (7%)
22	Neutrophil-to-lymphocyte ratio (NLR)	0.42	23	3.33 (2.3)	29 (7%)
23	Monocyte (%)	0.8	19.2	7.62 (2.5)	28 (7%)
24	Eosinophil (%)	0	23	2.5 (2.5)	28 (7%)
25	Basophil (%)	0	3.5	0.64 (0.36)	29 (7%)
26	Blood urea nitrogen (BUN, mg/dL)	3	54.4	13.9 (5.6)	11 (3%)
27	Creatinine (mg/dL)	0.07	15	0.94 (0.9)	4 (1%)
28	eGFR (mL/min/ 1.73 m^2^)	0.7	232.2	95.5 (30.8)	6 (1%)
29	GOT (U/L)	7	141	21.6 (13.7)	36 (9%)
30	GPT (U/L)	4	160	20.4 (15.8)	67 (16%)
31	Sodium (Na, mEq/L)	129	145	138.9 (2.7)	41 (10%)
32	Potassium (K, mEq/L)	2.6	5.6	3.92 (0.45)	40 (10%)
33	Carcinoembryonic antigen (CEA, ng/mL)	0.17	559.71	17.2 (44.4)	25 (6%)

### 3.2 Model Performance

Tables 3 and 4 show that all four models achieved accuracy above 70%. In the nCCRT plus CT group, the RC model performed best, with an accuracy of 77.3%, precision of 77.3%, and an AUC of 0.84. In the nCCRT group, the K-Star model attained the highest performance, with an accuracy of 80.8%, precision of 80.9%, and an AUC of 0.89. Overall, the models in the nCCRT group demonstrated slightly better predictive performance compared to those in the nCCRT plus CT group.

**Table 3:** Performance of machine learning models in predicting improvement among patients treated with neoadjuvant concurrent chemoradiotherapy (nCCRT) plus chemotherapy (CT)

Algorithm	Accuracy, % (95%CI)	Precision, %	Recall, %	F1, %	AUROC
K-Star	74.5 (70.0–78.4)	74.4	74.4	74.4	0.82 (0.77–0.88)
RF	74.7(70.2–78.6)	74.7	74.7	74.7	0.84 (0.79–0.89)
MLP	70.3(65.7–74.5)	70.3	70.3	70.3	0.74 (0.67–0.80)
RC	77.3(73.0–81.1)	77.3	77.3	77.3	0.84 (0.79–0.89)

Note: Random forest, RF; Multilayer perceptron, MLP; Random committee, RC; Area under the receiver operating characteristic curve, AUROC.

**Table 4:** Performance of machine learning models in predicting improvement among patients treated with neoadjuvant concurrent chemoradiotherapy (nCCRT) only

Algorithm	Accuracy, % (95%CI)	Precision, %	Recall, %	F1, %	AUROC
K-Star	80.8 (76.7–84.3)	80.9	80.8	80.8	0.89 (0.83–0.94)
RF	79.9 (75.8–83.5)	80.7	79.9	79.3	0.88 (0.82–0.93)
MLP	76.7 (72.3–80.5)	76.5	76.6	76.6	0.82 (0.76–0.89)
RC	74.7 (70.3–78.7)	74.5	74.7	74.4	0.83 (0.77–0.90)

Using ML-derived thresholds and attribute evaluators on the balanced dataset, cN consistently appeared as the top-ranked attribute across all methods (Table 5). Other frequently identified variables included cT, MCHC, platelet indices, and laboratory measures such as GPT, eGFR, and BUN.

**Table 5:** Top-ranked attributes identified by attribute ranking algorithms

Rank	Attribute ranking algorithm				
	Information gain	ReliefF	OneR	CfsSubset	Correlation
1	cN	cN	cN	cN	cN
2	MCHC	PLT	PDW	MCHC	Na
3	cT	Height	Lymphocyte (%)	N/A	GPT
4	Sex	WBC	GPT	N/A	eGFR
5	cM	Weight	BUN	N/A	Weight
6	N/A	eGFR	GOT	N/A	RBC
7	N/A	Age	MCHC	N/A	MCHC
8	N/A	Monocyte (%)	Eosinophil (%)	N/A	BUN

Note: N/A: Not Applicable.

Through consultation with clinicians, the correlation-based ranking was identified as the most clinically appropriate and explainable set of features. Clinicians noted that the highest-ranked attributes, cN, *sodium, GPT, eGFR, weight, RBC count, MCHC*, and *BUN*, are routinely considered during treatment planning and decision-making and represent clinically important markers in the management of rectal cancer.

ML models, such as the decision stump and AdaBoostM1 (aimed at deriving clinical thresholds), were restricted to patients who achieved improvement. We compared those treated with nCCRT plus CT against those treated with nCCRT alone. Several baseline thresholds emerged, differentiating between the two regimens. For patients who improved after receiving nCCRT plus CT, rules with high confidence included a lymphocyte percentage greater than 39.6% (99%), platelet distribution width (PDW) of ≤10.25 fL (85.7%), platelet counts above 394.0/µL (79%), and cN ≠ 2 (60.7%) (Table 6). In contrast, among patients who improved with nCCRT alone, the rules most often identified were cN = 2 (75.4%), estimated glomerular filtration rate (eGFR) above 140.10 mL/min/1.73 m^2^ (81%), and glutamic pyruvic transaminase (GPT) greater than 25.5 U/L (69%) (Table 7). These thresholds should be viewed as descriptive patterns for interpretability rather than clinical decision cutoffs. Taken together, these findings suggest that both tumor burden (nodal status) and hematologic and biochemical features differ between patients who benefitted from additional chemotherapy and those who improved with nCCRT alone.

**Table 6:** Machine learning (ML)-Derived thresholds for patients who received nCCRT plus CT

Technique	Rule	Confidence (%)
AdaBoostM1	LYM (%) > 39.6	99
DecisionStump	PDW (fL) ≤ 10.25	85.7
AdaBoostM1	PLT (/µL) > 394.0	79
AdaBoostM1	cN≠2	60.7

Note: LYM (%), Lymphocyte (%); PDW, Platelet distribution width; PLT, Platelet count; cN, Clinical N stage.

**Table 7:** ML-Derived thresholds for patients who received nCCRT alone

Technique	Rule	Confidence (%)
AdaBoostM1	eGFR (mL/min/1.73 m^2^) > 140.10	81
	cN = 2	75.4
	GPT (U/L) > 25.5	69

Note: eGFR, Estimated glomerular filtration rate; cN, Clinical N stage; GPT, Glutamic pyruvic transaminase.

## 4 Discussion

The most debated argument in the management of locally advanced rectal cancer (LARC) concerns the role and optimal timing of additional chemotherapy (CT) in relation to neoadjuvant concurrent chemoradiotherapy (nCCRT) and surgery. While nCCRT before surgery has become the standard approach owing to its proven benefits in tumor downstaging, sphincter preservation, and improved local control, the value of giving adjuvant CT after nCCRT and surgery remains uncertain. Some surgeons may incorporate chemotherapy before surgery to improve systemic control, whereas others suggest that adjuvant CT after surgery provides a limited survival advantage and may reduce patient compliance. Others may prefer nCCRT alone, which allows patients to have fewer surgical complications and improved postoperative recovery.

Chemotherapy is known to carry substantial risks, which can affect a patient’s quality of life. Therefore, identifying which patients benefit most from additional CT after nCCRT is clinically essential. In this study, both treatment groups, those receiving nCCRT alone and those receiving nCCRT with additional CT, were evaluated using ML models to predict improvement within each regimen. We compared the baseline demographics, tumor stages, renal and hepatic function, electrolytes, and hematologic features across both treatment groups, and explored potential patterns that may help explain differences in treatment response and serve as preliminary signals for future decision-support research.

One study has shown improvement in long-term survival after neoadjuvant treatment, which downstages the cancer stage and reduces the cancer burden in patients with breast cancer [22]. Thus, it is widely accepted in the clinical setting to treat patients according to their improved pathological cancer status. Therefore, we also investigated whether additional CT before surgery resulted in cancer downstaging. If additional CT can downstage cancer, an improved pathological cancer status could lead to better survival outcomes, which would be a significant benefit for patients. By contrast, delayed surgery or elevated surgical complications are the least desirable outcomes for both surgeons and patients. Surgery remains the most common treatment for most cancers, and complications associated with neoadjuvant therapies that delay or alter curative surgery are not worthwhile. This is another consideration for surgeons who prefer nCCRT alone.

Furthermore, some arguments suggest that patient selection in prospective randomised clinical trials (RCTs) may be biased and may not fully reflect clinical decision-making [23]. Clinical judgment encompasses more than scientific evidence; it also considers the individual condition of the patients. Research has concluded that real-world studies can complement data from RCTs by providing larger datasets, diverse patient populations, and quick responses to clinical dilemmas [24]. However, a more effective analytical tool for processing larger amounts of clinical data, minimising diverse clinical biases, and building robust prediction models is essential for current clinical research.

Based on demographic and clinical factors, most studies in the literature have focused on the use of ML models for early- or late-stage cancer detection. For instance, a related study from China, based on laboratory data from CRC patients and healthy individuals, revealed that a logistic regression model provided a powerful, non-invasive, and cost-effective method for identifying CRC, particularly late-stage colon cancer [15]. Another study from South Africa examined CRC recurrence and survival prediction, suggesting that statistical models should be used alongside ML models to enhance global interpretation and that two statistical models, logistic regression (LR) and naive Bayes (NB), are as significant as ML models such as RF, support vector machine (SVM), and artificial neural network (ANN) [25]. An Iranian study focused on metastasis prediction in CRC patients in a tertiary care facility and reported that neural networks (NNs) and RFs, followed by SVMs, were the best ML approaches with the highest AUC, sensitivity, and accuracy [26]. A Canadian study developed and validated a tool for the risk prediction of early discontinuation of adjuvant CT among patients with stage III colon cancer using a database from the Alberta government. The RF model may help in predicting early discontinuation of CT, in contrast to the LR model, which is less accurate in correctly classifying patients [27]. Another study [28] used various ML algorithms, including the best-performing model, logistic regression, and predicted survival estimation among patients with CRC. A retrospective study applied deep learning models to predict survival and assess the benefit of adjuvant chemotherapy in stage II/III CRC, highlighting the growing role of AI in treatment planning [29]. Additionally, deep distribution based multiple instance learning has been used for CRC survival prediction, showing the potential of advanced DL frameworks for outcome modeling [30]. Similarly, a Taiwanese study used different ML classifier models for colon cancer tumor staging and survival prediction via histopathological data and reported that the RF was the best predictor of the five-year disease-free survival of patients with colon cancer [13]. Compared to these findings, our study used similar demographic and clinical patient data but explored the application of ML models (K Star, RF, RC, and MLP) in decision support to assess their potential in guiding treatment choices for locally advanced rectal cancer. We developed two separate machine learning models for patients treated with nCCRT alone and those receiving additional chemotherapy. Across both regimens, the ML models demonstrated good predictive performance, with AUC values ranging from 0.74 to 0.89 and accuracies ranging from 70% to 81%. The K-Star model showed the strongest performance in the nCCRT group (AUC = 0.89), while the RC model performed best in the nCCRT plus CT group (AUC = 0.84). From a clinical perspective, these findings suggest that ML-based classifiers can meaningfully distinguish patients more likely to improve after nCCRT alone from those who may benefit from additional chemotherapy. Although these performance levels are not yet sufficient for independent clinical decision-making, they provide a useful early signal for decision support by helping to identify patients who may require closer clinical assessment or additional treatment consideration. These findings demonstrate that even with a modest cohort size, baseline clinical and laboratory features contain informative patterns that can support early treatment assessment in LARC.

ML-based models such as Decision Stump and AdaBoostM1 further identified clinically interpretable thresholds. Patients who improved after nCCRT plus CT were characterised by higher lymphocyte percentages, higher platelet counts, and lower platelet distribution width, whereas those who improved after nCCRT alone tended to show higher nodal stage, elevated GPT, and higher eGFR. While preliminary, these insights highlight the potential of ML-based approaches to complement clinical judgment. If validated in larger cohorts, such models have the potential to guide treatment planning to minimise unnecessary chemotherapy exposure and optimise patient outcomes. Foundation models built using digital pathology have also shown promise for predicting prognosis and therapy benefit in gastrointestinal cancers [31].

Since their inception, the use of clinical decision support systems (CDSSs) has rapidly increased owing to their potential to collect and analyse information that can be used in clinical decision support [32]. The integration of AI (including ML, Deep learning, and Natural language processing) has propelled the CDSS into a new era by enhancing its data processing and interpretation capabilities in terms of speed and accuracy [33]. ML algorithms play a key role in non-knowledge-based CDSSs by enabling them to process and analyse large datasets (big data) to extract meaningful insights that healthcare providers can use to make intelligent decisions and accurate predictions for improved personalised care and patient outcomes. Most studies in the CDSS field involve rule-based CDSSs that do not represent real-world data and focus on predicting the onset of conditions. Thus, there are only a few studies in the field of nonknowledge-based CDSSs for cancer prediction [34]. Since the recurrence rate after CRC is high, periodic surveillance and selection of appropriate CT treatment recommendations are important for CRC patients [35–37]. A closely related study from Korea developed an EMR-based DL model, the colorectal cancer chemotherapy recommender (C3R), to provide CT recommendations for CRC patients [34]. The results showed that the AUC of the C3R model demonstrated excellent performance with EMR data from Gachon Gil Medical Center in Korea. Several variables contributed to the model’s output, with pathological variables such as TNM stage and tumor location being the most significant factors that influenced the model. Moreover, other demographic variables such as age, smoking history, and histological type also influenced the results. The highest-ranked features in our analysis also align with clinical considerations in LARC. Clinical N stage, which consistently emerged as the predominant variable, reflects the extent of nodal involvement and is routinely considered when discussing the potential role of adding chemotherapy to nCCRT. Several laboratory markers, including GPT, eGFR, sodium, RBC indices, and BUN, were also selected among the top features. Although not used as stand-alone predictors in practice, they capture aspects of baseline health and organ-function status that may relate to differing responses to nCCRT alone vs. nCCRT plus chemotherapy. The simple threshold-based rules in Table 5 illustrate how distinct ranges of these common laboratory values separated patients who improved under each treatment pathway. Taken together, these exploratory patterns provide a clinically coherent starting point for identifying which baseline characteristics may be associated with benefit from additional chemotherapy. For the future, these exploratory findings need an external validation in larger, prospective cohorts. Long-term studies are warranted to evaluate whether model-informed (AI-assisted) decision support improves patient survival and quality of life. Our study is among the few to apply machine learning to locally advanced rectal cancer. By modelling outcomes separately for patients treated with nCCRT alone and those receiving nCCRT plus CT, we could compare predictive performance across regimens and identify clinically interpretable patterns. Another advantage is the use of clinical and laboratory variables, which enhances the potential for real-world application if validated further. The exploratory ML approach provided interpretable threshold cut-offs for clinical use. As this was a predictive, exploratory analysis, the findings reflect associations between baseline features and improvement, and no causal interpretation is implied.

Our study has several limitations. The findings were derived from retrospective data with a limited sample size, although the data were obtained from three different hospitals in Taiwan. An external validation cohort was not available for this study, so model performance was estimated using internal 10-fold cross-validation to provide a stable assessment. Therefore, further retrospective studies or prospective studies from different regions are recommended to confirm generalisability. Finally, larger datasets in future work would also support the use of post-hoc interpretability tools such as SHapley Additive exPlanations.

## 5 Conclusion

This study demonstrates that machine learning models can effectively support individualised treatment decisions for patients with locally advanced rectal cancer following neoadjuvant concurrent chemoradiotherapy. The models indicated good classification across both treatment regimens, with accuracies ranging from 75% to 81% (AUC 0.82–0.89) for nCCRT alone and from 70% to 77% (AUC 0.74–0.84) for nCCRT plus CT. Among the evaluated algorithms, K-Star showed the highest predictive accuracy for patients treated with nCCRT alone, while the RC model performed best for those receiving additional chemotherapy. Key attributes, such as clinical N stage, sodium level, GPT, eGFR, and hematologic parameters, provide valuable biological insights into treatment response. ML-derived thresholds suggested that higher lymphocyte percentages, lower platelet distribution width, elevated platelet counts, and lower cN nodal stages were associated with improved outcomes after nCCRT plus CT. In contrast, higher eGFR, mildly elevated GPT, and cN nodal stage 2 characterised patients who responded well to nCCRT alone. These findings are exploratory but provide a useful foundation for developing future decision-support tools to aid individualised neoadjuvant therapy planning. Larger, externally validated studies are needed to confirm these results and assess their clinical impact.

## Data Availability

The data are not available due to ethical restrictions.
